# Dietary inflammatory index and the risk of cardiovascular disease and mortality: A prospective cohort study of Chinese community-dwelling older adults

**DOI:** 10.1016/j.jnha.2025.100624

**Published:** 2025-07-03

**Authors:** Shu-Yi Li, Zhi-Hui Lu, Jason C.S. Leung, Yi Su, Blanche W.M. Yu, Timothy C.Y. Kwok

**Affiliations:** a Department of Medicine and Therapeutics, Prince of Wales Hospital, The Chinese University of Hong Kong, Hong Kong, China; b School of Nursing, The Hong Kong Polytechnic University, Hong Kong, China; c Jockey Club Centre for Osteoporosis Care and Control, The Chinese University of Hong Kong, Hong Kong, China; d Department of Epidemiology and Biostatistics, Key Laboratory of Molecular Epidemiology of Hunan Province, School of Public Health, Hunan Normal University, Changsha, China; e Faculty and Planning Office, Faculty of Medicine, The Chinese University of Hong Kong, Hong Kong, China

**Keywords:** Dietary inflammatory index, Cardiovascular disease, Cardiovascular risk factors, Older adults, Prospective cohort

## Abstract

**Objectives:**

Inflammation is a contributory factor for cardiovascular disease (CVD), and diet can modulate inflammation. This study aimed to investigate the association between dietary inflammatory index (DII) and CVD outcomes, and the mediating roles of cardiovascular risk factors.

**Design:**

A prospective cohort study.

**Setting and participants:**

A total of 3,013 Chinese community-dwelling older adults aged ≥65 years without CVD were included between 2001 and 2003 in Hong Kong.

**Measurements:**

DII was calculated using a 280-item validated food frequency questionnaire. CVD outcomes included incident CVD, coronary heart disease (CHD), stroke, and CVD mortality, which were obtained from official records. Cox proportional hazards models examined the association between tertiles of DII and CVD outcomes. Mediation analysis explored the mediating roles of cardiovascular risk factors, including inflammatory biomarkers, impaired renal function, abnormal ankle-brachial index (ABI), obesity, central obesity, diabetes mellitus, and hypertension.

**Results:**

There were 263 CVD cases, 147 CHD cases, and 130 strokes during a median follow-up of 5.7 years. There were 277 CVD deaths during a median follow-up of 16.8 years. The highest tertile of DII was associated with increased risks of CVD incidence (HR: 1.43, 95% CI: 1.05–1.96) and CVD mortality (HR: 1.45, 95% CI: 1.03–2.03) compared with the lowest tertile. No significant associations were found for CHD and stroke. Impaired renal function, abnormal ABI, and hyperhomocysteinemia mediated the effects of a pro-inflammatory diet on CVD risk, with mediated proportions ranging from 3.68% to 7.78%.

**Conclusion:**

A pro-inflammatory diet increased the risks of CVD incidence and mortality, mediated by impaired renal function, abnormal ABI, and hyperhomocysteinemia.

## 1 Introduction

Cardiovascular disease (CVD) is the leading cause of death worldwide, accounting for 32% of deaths in 2019 [1]. From 1990 to 2019, the prevalence of CVD nearly doubled from 271 million to 523 million cases, and related deaths rose from 12.1 million to 18.6 million, substantially increasing healthcare costs [2]. Dietary risks are a major contributor to CVD, with 7.9 million diet-attributable annual CVD deaths and 188 million annual disability-adjusted life years (DALYs) [2]. Diet can play a significant role in the pathophysiology of CVD by modulating chronic inflammation [3]. Dietary inflammatory index (DII) is used to quantify inflammatory potential of a diet based on 45 food parameters and their effects on six inflammatory biomarkers [4]. Numerous studies have supported that a pro-inflammatory diet, as measured by DII, is associated with increased risks of CVD and mortality [[5], [6], [7]].

Neverthelss, the underlying mechanism between the association between a pro-inflammatory diet and CVD remains largely unclear [8]. Increasing evidence has suggested that some cardiovascular risk factors (e.g., obesity, diabetes, hypertensions, and impaired renal function) could potentially mediate the association between DII and CVD [6]. Previous studies have observed that the positive association between DII and CVD was more pronounced in individuals with these cardiovascular risk factors [[9], [10], [11], [12]]. Furthermore, these cardiovascular risk factors are highly related to inflammation [13,14], suggesting that inflammatory biomarkers may also serve as an indirect pathway by which a pro-inflammatory diet increases CVD risk [6]. However, the potential mediating effects of these factors on the association between DII and CVD have been rarely examined. Only limited cross-sectional studies and one prospective study have been conducted, with inconsistent findings across studies [[15], [16], [17]]. To address this gap, the present study included several inflammatory biomarkers and established cardiovascular risk factors as potential mediators. Specifically, high-sensitivity C-reactive protein (hs-CRP) is a key marker of systemic inflammation [13]; impaired renal function reflects chronic inflammation [18]; and vitamin D and homocysteine are both diet-sensitive biomarkers associated with inflammation and cardiovascular risk [19,20]. In addition, traditional cardiovascular risk factor (obesity, central obesity, diabetes, and hypertension) were considered potential mediators [21,22], along with the ankle-brachial index (ABI), a non-invasive marker of subclinical atherosclerosis [21,22]. These insights may help inform the development of DII-based prevention strategies for high-risk populations.

Therefore, this prospective cohort study aimed to investigate the associations between DII and CVD outcomes among Chinese community-dwelling older adults and examine whether cardiovascular risk factors and inflammatory biomarkers mediate the associations between DII and CVD outcomes.

## 2 Material and methods

### 2.1 Study participants

Data were derived from the Mr. OS and Ms. OS (Hong Kong) cohort, an ongoing community-based prospective cohort study. Two thousand men and two thousand women aged 65 years or above living in the local communities were recruited between August 2001 and March 2003 by recruitment notices and health talks. A stratified sampling method was used to ensure an equal number in each of the three age groups: 65–69, 70–74, and 75 years or older. Participants were excluded if they lacked dietary data and measurements (n = 7), had extremely high or low energy intake (<800 kcal/day or >4000 kcal/day for men; <500 kcal/day or >3500 kcal/day; n = 13) [23], or had CVD (n = 826) and cancer (n = 141) at baseline. The final sample for analysis included 3,013 participants. The study was approved by the Clinical Research Ethics Committee of a local institution. Each participant provided written informed consent.

### 2.2 Dietary inflammatory index (exposure)

Dietary data was collected through face-to-face interviews conducted by trained research staff at baseline. A local 280-item validated food frequency questionnaire was used to estimate dietary intake during the past year [24]. Energy and nutrients intakes were calculated based on the Chinese Medical Sciences Institute and McCance and Widdowson [25,26]. DII was calculated using the scoring algorithm derived from a comprehensive literature review of 45 food parameters and their effects on six inflammatory markers (IL-1β, IL-4, IL-6, IL-10, TNF-α and CRP) [4]. Briefly, DII scores were calculated by obtaining z-scores for each food parameter from a global database, transforming them into centered percentile scores, multiplying them by their corresponding literature-derived inflammatory effect scores, and summing these scores to get total DII scores. Food parameters, literature-derived inflammatory effect scores and dietary intake values from the global dataset used in the calculation of DII were shown in Supplementary Table S1 [4]. In this study, the DII was calculated using 30 out of 45 food parameters. These parameters included: (1) macronutrients: protein, carbohydrate, total fat, saturated fat, monounsaturated fatty acid, polyunsaturated fatty acid, and cholesterol; (2) vitamins: vitamin A, β-carotene, thiamin, riboflavin, niacin, vitamin B6, folic acid, vitamin B12, vitamin C, vitamin D, and vitamin E; (3) minerals: iron, magnesium, selenium, and zinc; and (4) other parameters: fiber, isoflavones, alcohol, caffeine, onion, pepper, green/black tea, and energy. A validated study by Shivappa et al. reported that reducing the number of food parameters from 45 to 28 did not degrade the predictive ability of DII [27]. Higher positive DII scores indicate a more pro-inflammatory diet, and lower negative scores indicate a more anti-inflammatory diet. The DII score was categorized into sex-specific tertile: anti-inflammatory (the lowest tertile), intermediate (the middle tertile), and pro-inflammatory (the highest tertile). The median values of the DII in each tertile group were as follows: −2.11, −0.83, and 0.38 for men; and −1.67, −0.23, and 1.29 for women.

### 2.3 CVD incidence and mortality (CVD outcomes)

Cardiovascular events, including ischemic heart disease, ischemic and hemorrhagic stroke, angina, acute myocardial infarction, heart failure, acute coronary syndrome, and transient ischemic attack, were obtained from the Clinical Management System (CMS) database of the Hong Kong Hospital Authority (HA). The CMS database covers more than 90% of all hospital admissions in Hong Kong [28]. Incident cardiovascular events for CVD, CHD, and stroke were ascertained from baseline to September 2008. Mortality data were obtained from the Hong Kong Government Death Registry, and the causes of death were recorded using ICD-10 codes. CVD mortality was ascertained as the death record under I00-I99 codes from baseline to June 2020.

### 2.4 Potential mediators

In this study, inflammatory biomarkers, including hs-CRP, renal function, vitamin D, and homocysteine, as well as cardiovascular risk factors, including obesity, central obesity, diabetes mellitus, hypertension and abnormal ABI, were considered potential mediators.

#### 2.4.1 Inflammatory biomarkers

Fasting venous samples were collected at baseline. Serum was separated within 3 h and stored at −80 °C for subsequent analyses. Serum hs-CRP was measured using the Vitros Fusion 5.1 enzyme-linked immunosorbent assay (Vitros Chemistry Products, Rochester, NY) and was conducted by PathLab Co. Ltd. (Council Bluffs, IA). The intra-assay and inter-assay coefficient of variations (CVs) for hs-CRP were 1.1–1.4 % and 3.7–6.2 %. Serum hs-CRP levels were categorized as higher (≥3.0 mg/L) or lower (<3.0 mg/L) levels [29]. Serum 25-hydroxyvitamin D (25[OH]D), homocysteine, and creatinine were measured by liquid chromatography-tandem mass spectrometry. The CVs were 4–7 % for 25[OH]D and 3–7 % for homocysteine and creatinine. Vitamin D deficiency was defined as serum 25[OH]D levels less than 50 nmol/L [30]. Adequate vitamin D levels were defined as serum 25[OH]D levels more than 50 nmol/L. Only 0.84% of participants had vitamin D levels exceeding 100 nmol/L, thus we cannot further subdivide the high vitamin D status group. Hyperhomocysteinemia was defined as serum homocysteine levels higher than 15 μmol/L [31]. The estimated glomerular filtration rate (eGFR) was calculated according to the Chronic Kidney Disease Epidemiology Collaboration creatinine equation based on serum creatinine, sex, and age [32]. Impaired renal function was defined as eGFR less than 60 mL/min/1.73 m^2^ [33].

#### 2.4.2 Cardiovascular risk factors

Body weight was measured at baseline using a Physician Beam Balance Scale (Healthometer, McCook, IL, USA) with a precision of 0.1 kg. Height was measured using a stadiometer (Holtain Ltd., Crosswell, UK) with a precision of 0.1 cm. BMI was calculated by dividing weight in kilograms by the square of height in meters. BMI was categorized as <18.5; 18.5–22.9; 23–27.4; ≥27.5 kg/m^2^ [34]. Obesity was defined as BMI ≥ 27.5 kg/m^2^. Waist circumference was measured using a measuring tape while participants stood erect, with measurements recorded to the nearest 0.1 cm. Central obesity was defined as waist circumference >90 cm for men and >80 cm for women [35].

Diabetes mellitus or hypertension was assessed through self-reported history of diabetes or hypertension and use of antidiabetic or antihypertensive drugs. Participants were asked by questionnaire to confirm: (1) Have you ever been diagnosed with diabetes mellitus or hypertension by your physician? (2) If yes, have you sought medical treatment for this condition? In addition, participants brought in all current medications and prescriptions, which were inspected and recorded.

Blood pressure at the right arm and both ankles in the supine position were measured with a standard mercury sphygmomanometer and an 8 MHz Doppler probe (Pocket Doppler Model 841-A, Parks Medical Electronics, Inc., ALOHA, OR, USA) after participant resting for 5 min. An appropriately sized cuff was placed over the right brachial artery and around each ankle (above the malleolus). Systolic pressure was measured sequentially: right brachial artery, right lower extremity, and left lower extremity. Two measurements were performed for each site. ABI was calculated for each leg by dividing the systolic blood pressure at the ankle by the systolic blood pressure in the arm. The lowest ABI of the two legs was used for analysis. Abnormal ABI was defined as <0.9 or >1.4, indicating peripheral arterial diseases or arterial stiffening [36].

### 2.5 Covariates

Covariates were selected based on previous studies and their relevance as potential confounders in the association between DII and CVD outcomes, consistent with prior publication using this cohort [28]. These included demographic characteristics, lifestyle factors, and traditional cardiovascular risk factors. A standardized and structured questionnaire was used to collect information on demographics (sex, age, education level, living alone), lifestyles (smoking status, alcohol consumption, physical activity and energy intake), medical history and medication use at baseline. The Physical Activity Scale of the Elderly (PASE), adapted for the Chinese population in Hong Kong, was used to assess physical activities in leisure, household, and occupation-related activity over a 7-day period [37]. In addition, the Geriatric Depression Scale (GDS) score was included as a covariate, as depressive symptoms may be associated with both dietary patterns and cardiovascular outcomes, potentially confounding the observed associations. A validated Chinese version of GDS was used to assess depression symptoms. The survey consists of 15 questions related to depression, each scored one point [38]. Higher scores indicate a greater likelihood of depression, with scores above eight defining the presence of depressive symptoms.

### 2.6 Statistical analysis

Differences in baseline characteristics across tertiles of DII were examined using analysis of variance (ANOVA) for normally distributed or normality-transformed variables, the Kruskal-Wallis test for nonparametric variables, and χ^2^ test for categorical variables. Mean ± standard deviation (SD) for continuous variables and percentages (%) for categorical variables were presented.

Cox proportional hazards models were used to estimate the associations between tertiles of DII with CVD incidence and mortality. Hazard ratios (HRs) and corresponding 95% confidence intervals (95% CIs) were reported. Model 1 was adjusted for sex (men; women) and age (continuous); Model 2 was additionally adjusted for baseline sociodemographic and lifestyle factors, including education level (no education; primary or below; secondary or above), living alone (yes; no); smoking (yes; no), alcohol drinking (yes; no), GDS score (continuous), physical activity (continuous), energy intake (continuous), BMI (<18.5; 18.5–22.9; 23–27.4; ≥27.5 kg/m^2^), diabetes mellitus (yes; no) and hypertension (yes; no). The analysis was repeated separately for men and women due to the potential sex differences in the association between tertiles of DII and CVD outcomes.

The mediating roles of these cardiovascular risk factors, including higher hs-CRP level, vitamin D deficiency, hyperhomocysteinemia, impaired renal function, abnormal ABI, obesity, central obesity, diabetes mellitus, and hypertension, in the associations between tertiles of DII and CVD outcomes were examined using mediation analysis. Two regression models were constructed: the logistic regression model to regress tertiles of DII (exposure) on cardiovascular risk factors (mediator), and the Cox proportional hazards model to estimate the effects of tertiles of DII and cardiovascular risk factors on the risks of CVD incidence and mortality (outcome) with adjustment for covariates (sex, age, education level, living alone, smoking, alcohol drinking, physical activity and energy intake). These two regressions were integrated to estimate the direct and indirect effects of tertiles of DII on the risks of CVD incidence and mortality. The proportion of the mediating effect was estimated via bootstrapping. The "mediation" package in R was used for the mediation analysis [39].

Sensitivity analyses were conducted to test the robustness of the associations between tertiles of DII and CVD outcomes and the mediating effects of these cardiovascular risk factors. Analyses were repeated after excluding CVD death events occurring within two years after baseline or with additionally adjustment for antihypertensive, antidiabetic, and lipid-lowering drugs.

All statistical analyses were performed by using STATA (StataCorp. 2021. Stata Statistical Software: Release 17. College Station, TX: StataCorp LLC) and RStudio (Posit team 2024. RStudio Version 2024.12.0+467: Integrated Development Environment for R. Posit Software, PBC, Boston, MA, USA). A two-sided *p-*value <0.05 was considered statistically significant.

## 3 Results

### 3.1 Participant characteristics

Among 3,013 community-living older adults in this study, the mean age was 72.4 (SD 5.2) years and 51.0% of participants were women at baseline. The mean BMI was 23.5 (SD 3.3) kg/m^2^. The mean DII was −0.48 (SD 1.46), and the median DII was −0.52 (IQR −1.50, 0.43). There were 263 CVD cases, including 147 CHD cases and 130 strokes during a median follow-up of 5.7 years. There were 277 CVD deaths during a median follow-up of 16.8 years. Baseline characteristics across tertiles of DII are shown in Table 1. Participants with higher DII score were more likely to be older, smokers, and live alone. They also had lower educational levels, higher GDS scores, lower physical activity, and lower energy intake, compared to those with lower DII scores (*p* < 0.05). In addition, participants with higher DII scores were more likely to have higher serum hs-CRP levels, abnormal ABI, hyperhomocysteinemia, and impaired renal function, compared with those with lower DII scores (*p* < 0.05). However, there were no significant differences in BMI, waist circumference, blood pressure, the prevalence of diabetes mellitus and hypertension, and serum 25[OH]D levels across tertiles of DII.

**Table 1:** Baseline characteristics of participants across tertiles of DII.

	Tertiles of DII			*p*
	T1 (n = 1,004)	T2 (n = 1,004)	T3 (n = 1,005)	
DII, score	−2.00 ± 0.71	−0.52 ± 0.47	1.09 ± 0.96	<0.001
Age, year	71.77 ± 5.00	72.34 ± 5.15	72.98 ± 5.43	<0.001
Men, %	49.00	49.00	48.96	1.000
Education, %				<0.001
No education	17.43	22.91	25.77	
Primary or below	49.00	48.90	52.54	
Secondary or above	33.57	28.19	21.69	
Living alone, %	8.86	10.46	13.13	0.008
Smoking, %	6.67	5.98	9.65	0.004
Alcohol drinking, %	17.23	15.04	9.05	<0.001
GDS score	2.54 ± 2.61	2.61 ± 2.47	3.25 ± 2.94	<0.001
Weight, kg	58.55 ± 9.70	58.04 ± 9.57	57.33 ± 9.54	0.017
BMI, kg/m^2^	23.59 ± 3.25	23.58 ± 3.20	23.39 ± 3.34	0.309
Obesity ^a^, %	10.96	9.86	10.55	0.720
Waist circumference, cm	85.67 ± 9.24	86.05 ± 8.88	86.14 ± 9.55	0.482
Central obesity ^b^, %	52.99	55.58	57.01	0.184
ABI	1.08 ± 0.16	1.08 ± 0.13	1.08 ± 0.23	0.576
Abnormal ABI ^c^, %	6.37	7.47	9.95	0.010
Hypertension, %	41.18	38.94	44.18	0.057
Diabetes mellitus, %	12.66	12.85	13.43	0.866
PASE score	98.53 ± 46.15	92.06 ± 42.99	85.75 ± 40.12	<0.001
Energy intake, kcal/d	2140.84 ± 570.27	1803.82 ± 509.73	1557.77 ± 478.22	<0.001
Carbohydrate intake, g/d	298.08 ± 85.16	249.74 ± 74.91	221.27 ± 71.07	<0.001
Protein intake, g/d	95.72 ± 34.86	73.91 ± 27.62	57.56 ± 22.90	<0.001
Fat intake, g/d	65.52 ± 22.78	57.95 ± 22.70	49.86 ± 22.25	<0.001
Serum hs-CRP ^d^, mg/L	2.97 ± 5.53	3.71 ± 9.68	4.05 ± 10.03	0.017
Higher hs-CRP level ^e^, %	23.22	30.92	33.83	<0.001
Serum 25[OH]D ^f^, nmol/L	59.57 ± 15.44	59.88 ± 15.33	58.64 ± 14.67	0.528
Vitamin D deficiency ^g^, %	30.49	28.81	29.97	0.700
Serum homocysteine ^h^, μmol/L	13.71 ± 4.23	13.88 ± 4.17	14.51 ± 4.90	0.001
Hyperhomocysteinemia ^i^, %	27.08	31.09	35.24	0.003
eGFR ^h^, mL/min/1.73 m^2^	83.00 ± 13.08	81.93 ± 13.41	79.49 ± 15.01	<0.001
Impaired renal function ^j^, %	6.96	7.64	12.79	<0.001

Abbreviation: DII, dietary inflammatory index; T1, the first tertile; T2, the second tertile; T3, the third tertile; GDS, geriatric depression scale; BMI, body mass index; SBP, systolic blood pressure; DBP, diastolic blood pressure; ABI, ankle-brachial index; PASE, Physical Activity Scale for the Elderly; hs-CRP, high-sensitivity C-reactive protein; 25[OH]D, 25-hydroxyvitamin D; eGFR, estimated glomerular filtration rate.

Mean ± Standard Deviation for continuous variables; percentage (%) for categorical variables.

^a^Obesity was defined as BMI ≥ 27.5 kg/m^2^; ^b^ central obesity was defined as waist circumference >90 cm for men or >80 cm for women; ^c^ Abnormal ABI was defined as ABI < 0.9 or ABI > 1.4; ^d^ Available in 1,076 women and 1,069 men; ^e^ higher hs-CRP level was defined as serum hs-CRP ≥ 3.0 mg/L; ^f^ Available in 1,102 women and 1,053 men; ^g^ Vitamin D deficiency was defined as serum 25[OH]D <50 nmol/L; ^h^ Available in 1,217 women and 1,082 men; ^i^ Hyperhomocysteinemia was defined as serum homocysteine ≥15 μmol/L; ^j^ Impaired renal function was defined as eGFR <60 mL/min/1.73 m^2^.

### 3.2 Associations between DII and CVD outcomes

**Fig. 1:**
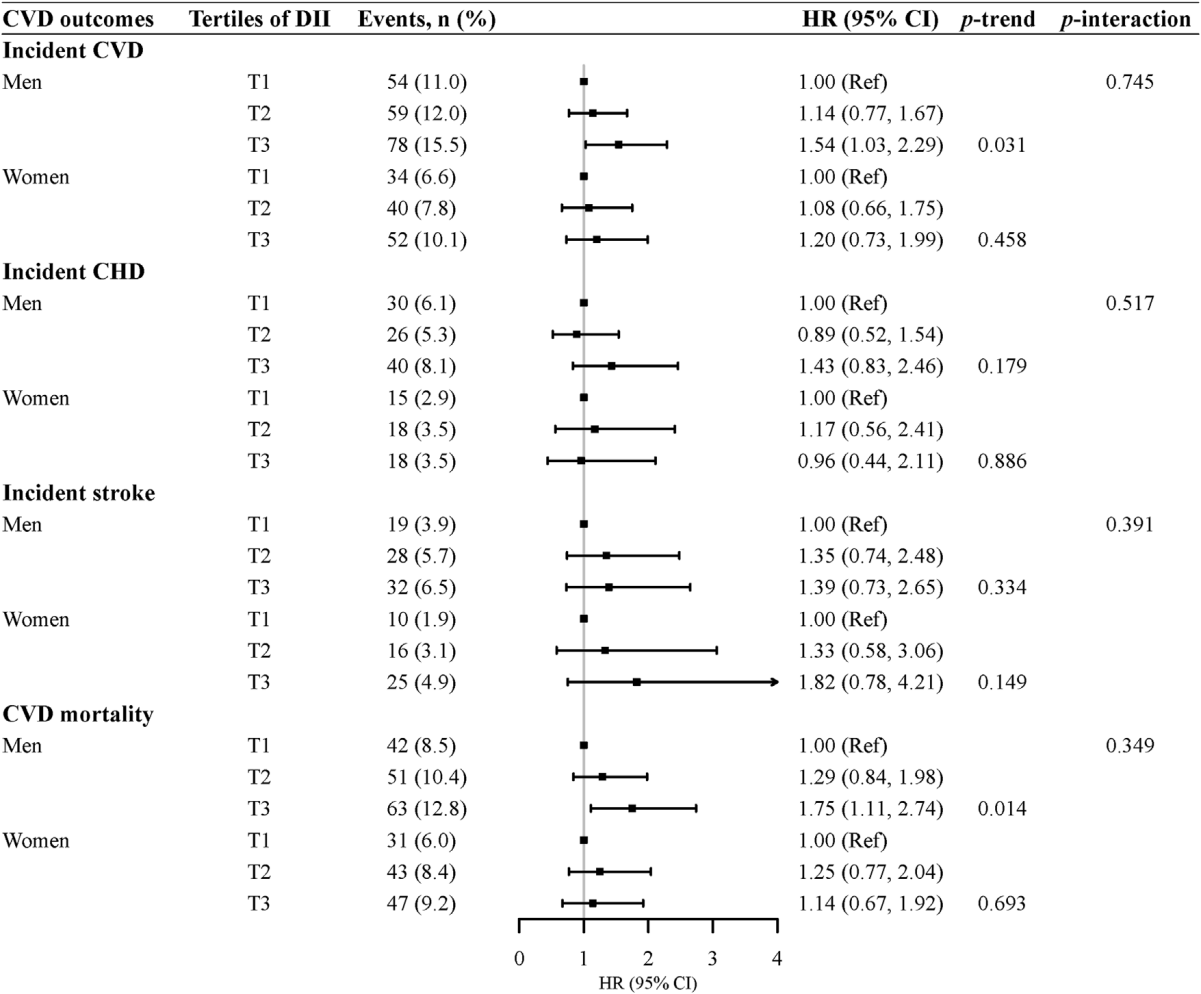
Hazard ratios and 95% confidence intervals for the association between DII and CVD outcomes stratified by sex. Abbreviation: DII, dietary inflammatory index; CVD, cardiovascular disease; CHD, coronary heart disease; HR, hazard ratio; 95% CI: 95% confidence interval; Ref, reference. Adjusted for age, education level, living alone; smoking, alcohol drinking, GDS score, physical activity, energy intake, BMI, diabetes mellitus and hypertension.

Associations between tertiles of DII and CVD outcomes are presented in Table 2. In the age- and sex-adjusted model, the highest tertile of DII was significantly associated with increased risks of incident CVD (HR: 1.40; 95% CI: 1.07, 1.84; *p*-trend = 0.012), incident stroke (HR: 1.91; 95% CI: 1.22, 2.99; *p*-trend = 0.005), and CVD mortality (HR: 1.59; 95% CI: 1.18, 1.77; *p*-trend = 0.002), compared with the lowest tertile of DII. The positive association between DII and incident CVD did not change, but the risk for CVD mortality was attenuated after adjustment for baseline sociodemographic and lifestyle factors, with HRs of 1.45 (95% CI: 1.03, 2.03) when compared the highest tertile of DII with the lowest. In addition, the association between DII and incident stroke became insignificant (HR: 1.56; 95% CI: 0.94, 2.59) in the multivariate-adjusted model. No significant association was found between tertiles of DII and incident CHD. The positive associations between DII and risks of CVD incidence and mortality were found in older men, but no significant association was found in older women (Fig. 1). However, there was no significant interaction between tertiles of DII and sex on CVD risk and mortality (Fig. 1).

**Table 2:** Associations between tertiles of DII and CVD outcomes.

	Tertiles of DII, HR (95% CI)			*p*-trend a
	T1	T2	T3	
Incident CVD				
No. case	88	99	130	
Person years	5488	5414	5251	
Model 1^b^	1.00 (Ref)	1.09 (0.82, 1.46)	1.40 (1.07, 1.84)	0.012
Model 2^c^	1.00 (Ref)	1.14 (0.85, 1.54)	1.43 (1.05, 1.96)	0.020
Incident CHD				
No. case	45	44	58	
Person years	5611	5563	5438	
Model 1	1.00 (Ref)	0.96 (0.63, 1.45)	1.23 (0.83, 1.45)	0.273
Model 2	1.00 (Ref)	1.02 (0.66, 1.58)	1.33 (0.85, 2.07)	0.203
Incident Stroke				
No. case	29	44	57	
Person years	5645	5560	5439	
Model 1	1.00 (Ref)	1.49 (0.93, 2.38)	1.91 (1.22, 2.99)	0.005
Model 2	1.00 (Ref)	1.36 (0.83, 2.21)	1.56 (0.94, 2.59)	0.090
CVD mortality				
No. case	73	94	110	
Person years	15239	14588	13861	
Model 1	1.00 (Ref)	1.31 (0.96, 1.77)	1.59 (1.18, 1.77)	0.002
Model 2	1.00 (Ref)	1.27 (0.93, 1.76)	1.45 (1.03, 2.03)	0.034

Abbreviation: DII, dietary inflammatory index; CVD, cardiovascular disease; CHD, coronary heart disease; HR, hazard ratio; 95% CI: 95% confidence interval; Ref, reference.

a *p* for trend was calculated by treating the median values of DII in tertiles as continuous values in Cox regression models.

b Model 1: adjusted for sex, age.

c Model 2: adjusted for model 1 plus education level, living alone; smoking, alcohol drinking, GDS score, physical activity, energy intake, BMI, diabetes mellitus and hypertension.

### 3.3 The mediating effects of cardiovascular risk factors in associations between DII and CVD outcomes

In mediation analysis, impaired renal function, abnormal ABI, and hyperhomocysteinemia mediated the positive association between DII and CVD incidence by 6.01%, 3.68%, and 7.54%, respectively (Table 3). Moreover, impaired renal function, abnormal ABI, and hyperhomocysteinemia accounted for 6.84%, 5.16% and 7.78% of the association between DII and CVD mortality, respectively. No significant mediating effects were observed for higher hs-CRP levels, vitamin D deficiency, obesity, central obesity, diabetes mellitus, and hypertension. (Table 3). Associations between inflammatory biomarkers and cardiovascular risk factors with CVD outcomes were shown in Supplementary Table S2.

**Table 3:** The proportion mediated by cardiovascular risk factors on the associations between tertiles of DII and risks of CVD incidence and mortality.^a^

	CVD incidence		CVD mortality	
	Proportion mediated, %	*p*	Proportion mediated, %	*p*
Higher hs-CRP level	2.99 (−1.59, 15.8)	0.194	2.32 (−1.80, 12.21)	0.238
Hyperhomocysteinemia	7.54 (2.62, 22.89)	<0.001	7.78 (2.48, 27.59)	<0.001
Vitamin D deficiency	0.03 (−8.74, 8.01)	0.966	0.01 (−11.68, 6.98)	0.994
Impaired renal function	6.01 (2.02, 19.81)	0.008	6.84 (2.24, 23.36)	0.008
Abnormal ABI	3.68 (0.59, 11.27)	0.018	5.16 (1.02, 13.50)	0.014
Obesity	−0.09 (−3.28, 1.81)	0.814	−0.28 (−3.90, 1.45)	0.580
Central obesity	0.28 (−4.91, 5.64)	0.872	0.06 (−1.74, 1.78)	0.964
Diabetes mellitus	0.39 (−5.73, 6.23)	0.838	0.37 (−5.89, 6.25)	0.840
Hypertension	3.22 (−4.39, 12.24)	0.346	1.07 (−1.78, 6.44)	0.372

Abbreviation: DII, dietary inflammatory index; CVD, cardiovascular disease; hs-CRP, high-sensitivity C-reactive protein; ABI, ankle-brachial index.

a Mediated proportions and 95% confidence intervals were presented. Adjusted for sex, age, education level, living alone, smoking, alcohol drinking, physical activity, and energy intake.

### 3.4 Sensitivity analysis

In sensitivity analyses, the associations between DII and CVD outcomes remained unchanged after adjusting for antihypertensive, antidiabetic, and lipid-lowering drugs, and after excluding CVD deaths within two years (Supplementary Table S3). Moreover, the mediating roles of impaired renal function, abnormal ABI and hyperhomocysteinemia in the association between DII and CVD incidence and mortality remained unchanged (Supplementary Table S4).

## 4 Discussion

In this prospective cohort study of community-dwelling older adults, higher DII scores, indicating a more pro-inflammatory diet, were associated with increased risks of developing CVD and CVD mortality. Furthermore, the positive association between DII and CVD outcomes was mediated by impaired renal function, abnormal ABI, and hyperhomocysteinemia.

This study calculated DII scores based on the methodology developed by Shivappa et al. [4], using 30 food parameters derived from a validated FFQ. This method is consistent with many previous studies, although the dietary assessment tools (including 24-h dietary recalls, FFQ, and food records) and the number of food components (ranging from 20 to 37) used across studies vary considerably (Supplementary Table S5). These differences may influence the resulting DII scores. For instance, more detailed tools like food records and a greater number of food components may produce more accurate scores. In this Hong Kong elderly cohort, the mean DII score was −0.48 (SD 1.46), which is higher than that reported in a cohort from mainland China (−0.8 ± 1.1) [15] and a Spanish cohort (−0.75 ± 1.53) [40], but lower than those observed in cohorts from the United States (1.43 ± 1.86) [16], Germany (0.89 ± 1.38) [41], and South Korea (Median 0.92 for men and 0.89 for women) [42]. A Japanese cohort study of 58,782 participants aged 40–79 years reported a mean DII of 1.02, using 26 parameters from FFQ [43], while another Japanese cohort of 9284 participants aged 30–92 years reported −0.44, using 20 parameters from 3-day food records [44]. This supports that methodological differences in DII estimation may contribute differences in DII values. However, both studies observed a positive association between DII and CVD mortality. Despite variations in DII values, most studies, including this study, reported positive associations between higher DII scores and increased risk of total CVD [7]. However, findings for specific CVD outcomes remain inconsistent [42,[45], [46], [47]]. For example, a meta-analysis of 14 observational studies showed that a pro-inflammatory diet increased the risk of total CVD and myocardial infarction, but it was not associated with stroke or ischemic heart disease in subgroup analyses [5]. A Korean cohort study of 162,773 participants aged 40–79 years found a positive association between DII (37 parameters from FFQ) and total CVD but not with myocardial infarction or stroke during a 7.4-year follow-up [42]. A prospective cohort study involving 6,972 middle-aged Australian women found no significant association between DII (25 parameters from FFQ) and risks of total CVD, ischemic heart disease, myocardial infarction, cerebrovascular disease or stroke over an 11-year follow-up [45]. Besides, findings from three large U.S. prospective cohort studies reported that higher empirical dietary inflammatory pattern scores, which is based on food groups rather than nutrients, were associated with increased incidences of CVD, CHD, and stroke over 24–30 years of follow-up [47]. Methodological differences in dietary assessment, the number of food parameters included in DII calculation, and population dietary patterns may influence DII scores and potentially affect the observed associations. Moreover, differences in study populations and follow-up durations may also contribute to the heterogeneity of findings across studies. In this study, no significant association was observed between DII and incident CHD or stroke. This may be due to the low incidence of CHD and stroke, which may not provide sufficient statistical power. More studies are needed to investigate the association between a pro-inflammatory diet and various types of CVD, which could inform dietary recommendations for preventing or managing specific CVD conditions.

Although no interaction between DII and sex on CVD outcomes was found, a pro-inflammatory diet was found to increase CVD risk in older men but not in women in this study. This result was consistent with a Korean cohort study of 162,773 participants aged 40–79 and a Chinese cohort study of 4,822 adults [15,42]. In contrast, a meta-analysis of observational studies found increased CVD risk only in women when comparing the highest DII category with the lowest category. This discrepancy may be due to the limited number of studies on men (two studies) versus women (five studies) in the subgroup analysis [5]. According to the Global Burden of Disease study, men showed higher numbers and proportions of diet-related CVD deaths and DALYs, indicating greater susceptibility to unhealthy diets [48]. Furthermore, sex differences in CVD risk are influenced by hormones, lipid metabolism, inflammation response, and gene expression [49]. For example, estrogen can lower low-density lipoprotein cholesterol and inflammatory markers, protecting the cardiovascular health of women, while testosterone can increase these levels in men, promoting atherosclerosis and stroke [49]. However, this protective effect of estrogen diminishes after menopause. Notably, higher DII scores were significantly associated with higher levels of serum hs-CRP in men but not in women in this study (Data not shown). More studies are needed to explore the sex differences further.

Impaired renal function, abnormal ABI, and hyperhomocysteinemia may serve as potential pathways through which a pro-inflammatory diet increases the risks of developing CVD and CVD mortality. For impaired renal function, a community-based study of elderly Swedish individuals found that a pro-inflammatory diet was associated with decreased renal function, with serum CRP mediating this association [50]. Impaired renal function, a hallmark of chronic kidney disease, is strongly associated with CVD risk, particularly severe types of CVD outcomes such as CVD mortality [51]. As for ABI, it is a reliable and non-invasive measurement for peripheral arterial diseases and has been increasingly recognized as a marker of general atherosclerosis and cardiovascular risk [52]. Abnormal ABI increased more than doubles the 10-year rates of CVD events [36]. Our previous studies in the same participants observed that dietary patterns emphasizing fruit and vegetable intakes were associated with improved vascular health, as measured by ABI [53]. The protective effects of fruits and vegetables on vascular health may be due to their rich anti-inflammatory and antioxidant properties [54]. Thus, an anti-inflammatory diet may contribute to maintaining normal ABI, thereby decreasing CVD risk. For hyperhomocysteinemia, experimental studies have shown that homocysteine promotes endothelial dysfunction and prothrombotic effects, and several observational studies have shown that hyperhomocysteinemia is associated with higher CVD risk [31]. Active forms of folate, vitamin B12, vitamin B6, and riboflavin are involved in the metabolism of homocysteine [55]. These vitamins are included in the calculation of DII as dietary parameters [4]. A pro-inflammatory diet may elevate homocysteine levels by affecting these nutrients, thereby increasing CVD risk. Despite this, the mediation analysis revealed a percentage range of only 3.68%–7.78%. Moreover, no mediating effects were observed for hs-CRP, obesity, central obesity, diabetes or hypertension in the association between DII and CVD. Several factors may contribute to the weak or non-significant mediation effects observed in this study. First, other unmeasured mediating factors may influence the association between a pro-inflammatory diet and CVD outcomes, but may not have been captured in this study, such as blood lipid profiles or endothelial function [6]. The low prevalence of certain mediators, such as impaired renal function and abnormal ABI (around 7%), may have reduced statistical power to detect mediation. Moreover, both dietary data and mediators were measured only at baseline, restricting the ability to assess temporal relationships and causal mediation. It is possible that individuals with obesity, diabetes or hypertension are likely to receive dietary advice from healthcare providers, which may lead them to adjust their dietary patterns and other lifestyles either before inclusion in this study or during follow-up, potentially attenuating mediation effects. hs-CRP is commonly used in assessing inflammatory status, but it alone and only one time point measurement cannot fully characterize the complexity of immune system [44]. Besides, no mediating effect of vitamin D deficiency could be attributed to the limited impact of diet on vitamin D [56]. Given the complexity of interactions between diet, inflammation, and CVD, further studies are needed to investigate additional pathways using repeated measures, longer follow-up, and a broader range of biomarkers.

The strengths of this prospective cohort study include a relatively large sample size of older adults, reliable cardiovascular events and death data derived from the official database. In addition, this study used a validated assessment to evaluate the habitual dietary inflammatory potential and examined several known cardiovascular risk factors to identify potential mechanistic mediators in the associations between DII and CVD outcomes. Our findings revealed potential pathways through which a pro-inflammatory diet increases CVD risk, which may be helpful for the development of a mechanism-based dietary intervention strategy for the prevention and management of CVD. However, there are several limitations in this study. First, dietary data were collected only at baseline. We cannot analyze the changes in DII over time and their impact on CVD outcomes. Second, there was no data on anti-inflammatory dietary supplements, which could potentially interfere with the association between DII and CVD outcomes. Third, other cardiovascular risk factors (such as lipid profile) and other key inflammatory biomarkers (such as TNF-α and IL-6) were not measured in this study. Future research is needed to incorporate a broader range of inflammatory markers and cardiovascular risk factors to better understand the mechanisms linking dietary inflammation and CVD. Fourth, although the public hospital database covers over 90% of all hospital admissions in Hong Kong [28], this study may not have captured all CVD events during follow-up, as a small proportion of cases from private hospitals were not accessible. In addition, the relatively short follow-up period up to 7 years may have limited the number of CVD events identified. However, CVD mortality was determined using the Government Death Registry, which offers reliable long-term data up to 18 years follow-up and supports the results. Fifth, both dietary data and mediators were measured only at baseline, which may limit the ability to detect causal relationships. More studies are warranted to examine the potential pathways between dietary inflammation and CVD outcomes with repeated measures and long-term follow-up. Finally, participants in this study had higher education levels, health consciousness, and physical activity levels than the general Hong Kong population. This may limit the generalizability of the findings to other populations.

## 5 Conclusions

This prospective cohort study suggests that a pro-inflammatory diet increased the risks of CVD incidence and mortality in Chinese community-dwelling older adults. Impaired renal function, abnormal ABI, and hyperhomocysteinemia mediated the positive association between DII and CVD outcomes. For individuals with these high-risk conditions, adherence to an anti-inflammatory diet may be an effective strategy for early CVD prevention. Further research is needed to explore the mediating roles of cardiovascular risk factors, providing insights into the potential mechanisms linking diet, inflammation, and CVD.

## CRediT authorship contribution statement

Conceptualization, TCY Kwok; Methodology, TCY Kwok, ZH Lu, JCS Leung, Y Su and BWM Yu; Formal Analysis, SY Li and JCS Leung,; Resources, TCY Kwok; Writing—Original Draft Preparation, SY Li; Writing—Review and Editing, ZH Lu, JCS Leung, Y Su, BWM Yu and TCY Kwok; Supervision, TCY Kwok; Project Administration, TCY Kwok; Funding Acquisition, TCY Kwok. All authors had read and approved the final version of this manuscript.

## Ethics declarations

The study was conducted according to the guidelines of the Declaration of Helsinki, and approved by the Joint Chinese University of Hong Kong—New Territories East Cluster Clinical Research Ethics Committee (protocol code is CRE-2003.102 and it was renewed on 27 August 2005). Written informed consent was obtained from each participant in this study.

## Declaration of Generative AI and AI-assisted technologies in the writing process

During the preparation of this work, the authors used Microsoft Copilot to improve readability and enhance the clarity of the manuscript. After using this tool, the authors carefully reviewed and edited the content to ensure accuracy and take full responsibility for the content of this publication.

## Funding

This study was supported by the National Institutes of Health R01 grant [AR049439–01A1] and the Research Grants Council Earmarked grant [CUHK4101/02M].

## Data statement

The data that supports the findings of this study are available from the corresponding author upon reasonable request.

## Declaration of competing interest

The authors declare that they have no conflicts of interest.
