# Supplementary material for: Dietary inflammatory index and the risk of cardiovascular disease and mortality: A prospective cohort study of Chinese community-dwelling older adults
Source: J Nutr Health Aging. 2025 Jul 3;29(9):100624. doi: 10.1016/j.jnha.2025.100624 (PMC12271065; doi:10.1016/j.jnha.2025.100624)
Supplement: Supplementary file 1 [file mmc1.docx]

**Table S1**. Food parameters, inflammatory effect scores and dietary intake values from the global composite dataset in the calculation of DII [1].

| Food parameters | Literature-based inflammatory effect score | Global daily average intake (units/day) | SD |
| --- | --- | --- | --- |
| Energy (kcal) | 0.180 | 2056 | 338 |
| Carbohydrate (g) | 0.097 | 272.2 | 40.0 |
| Protein (g) | 0.021 | 79.4 | 13.9 |
| Total fat (g) | 0.298 | 71.4 | 19.4 |
| Saturated fat (g) | 0.373 | 28.6 | 8.0 |
| Monounsaturated fat (g) | −0.009 | 27.0 | 6.1 |
| Polyunsaturated fat (g) | −0.337 | 13.88 | 3.76 |
| n–3 Fatty acids (g) | −0.436 | 1.06 | 1.06 |
| n–6 Fatty acids (g) | −0.159 | 10.80 | 7.50 |
| Cholesterol (mg) | 0.110 | 279.4 | 51.2 |
| Trans fat (g) | 0.229 | 3.15 | 3.75 |
| Fiber (g) | −0.663 | 18.8 | 4.9 |
| Vitamin A (RE) | −0.401 | 983.9 | 518.6 |
| β-Carotene (µg) | −0.584 | 3718 | 1720 |
| Thiamin (mg) | −0.098 | 1.70 | 0.66 |
| Riboflavin (mg) | −0.068 | 1.70 | 0.79 |
| Niacin (mg) | −0.246 | 25.90 | 11.77 |
| Folic acid (µg) | −0.190 | 273.0 | 70.7 |
| Vitamin B_6_ (mg) | −0.365 | 1.47 | 0.74 |
| Vitamin B_12_ (µg) | 0.106 | 5.15 | 2.70 |
| Vitamin C (mg) | −0.424 | 118.2 | 43.46 |
| Vitamin D (µg) | −0.446 | 6.26 | 2.21 |
| Vitamin E (mg) | −0.419 | 8.73 | 1.49 |
| Iron (mg) | 0.032 | 13.35 | 3.71 |
| Zinc (mg) | −0.313 | 9.84 | 2.19 |
| Selenium (µg) | −0.191 | 67.0 | 25.1 |
| Magnesium (mg) | −0.484 | 310.1 | 139.4 |
| Eugenol (mg) | −0.140 | 0.01 | 0.08 |
| Turmeric (mg) | −0.785 | 533.6 | 754.3 |
| Flavan-3-ol (mg) | −0.415 | 95.8 | 85.9 |
| Flavones (mg) | −0.616 | 1.55 | 0.07 |
| Flavonols (mg) | −0.467 | 17.70 | 6.79 |
| Flavonones (mg) | −0.250 | 11.70 | 3.82 |
| Anthocyanidins (mg) | −0.131 | 18.05 | 21.14 |
| Isoflavones (mg) | −0.593 | 1.20 | 0.20 |
| Garlic (g) | −0.412 | 4.35 | 2.90 |
| Ginger (g) | −0.453 | 59.0 | 63.2 |
| Onion (g) | −0.301 | 35.9 | 18.4 |
| Pepper (g) | −0.131 | 10.00 | 7.07 |
| Thyme/oregano (mg) | −0.102 | 0.33 | 0.99 |
| Rosemary (mg) | −0.013 | 1.00 | 15.00 |
| Saffron (g) | −0.140 | 0.37 | 1.78 |
| Alcohol (g) | −0.278 | 13.98 | 3.72 |
| Caffeine (g) | −0.110 | 8.05 | 6.67 |
| Green/black tea (g) | −0.536 | 1.69 | 1.53 |

**Table S2**. Associations between cardiovascular risk factors and CVD outcomes.

|  | Model 1 ^a^ | | Model 2 ^b^ | |
| --- | --- | --- | --- | --- |
|  | HR (95% CI) | *p* | HR (95% CI) | *p* |
| **Incident CVD** |  |  |  |  |
| Higher hs-CRP level | 1.26 (0.96, 1.65) | 0.092 | 1.14 (0.86, 1.50) | 0.384 |
| Hyperhomocysteinemia | 1.56 (1.17, 2.09) | 0.002 | 1.50 (1.12, 2.02) | 0.006 |
| Vitamin D deficiency | 1.21 (0.92, 1.60) | 0.169 | 1.18 (0.89, 1.56) | 0.254 |
| Impaired renal function | 1.95 (1.35, 2.82) | <0.001 | 1.61 (1.11, 2.35) | 0.013 |
| Abnormal ABI | 1.63 (1.17, 2.29) | 0.004 | 1.57 (1.12, 2.20) | 0.009 |
| Obesity | 1.26 (0.88, 1.79) | 0.207 | 1.12 (0.79, 1.60) | 0.530 |
| Central obesity | 1.68 (1.32, 2.13) | <0.001 | 1.49 (1.17, 1.91) | 0.001 |
| Diabetes mellitus | 1.77 (1.35, 2.32) | <0.001 | 1.71 (1.30, 2.26) | <0.001 |
| Hypertension | 1.96 (1.57, 2.46) | <0.001 | 1.93 (1.53, 2.43) | <0.001 |
| **Incident CHD** |  |  |  |  |
| Higher hs-CRP level | 1.34 (0.89, 2,92) | 0.166 | 1.26 (0.83, 1.92) | 0.281 |
| Hyperhomocysteinemia | 1.47 (0.97, 2.21) | 0.068 | 1.46 (0.96, 2.22) | 0.076 |
| Vitamin D deficiency | 1.36 (0.89, 2.07) | 0.153 | 1.38 (0.89, 2.11) | 0.147 |
| Impaired renal function | 1.26 (0.70, 2.27) | 0.437 | 1.08 (0.59, 1.96) | 0.804 |
| Abnormal ABI | 1.82 (1.13, 2.94) | 0.014 | 1.73 (1.06, 2.80) | 0.027 |
| Obesity | 1.14 (0.67, 1.96) | 0.625 | 1.00 (0.58, 1.72) | 0.996 |
| Central obesity | 1.50 (1.06, 2.12) | 0.022 | 1.30 (0.91, 1.85) | 0.146 |
| Diabetes mellitus | 1.79 (1.20, 2.66) | 0.004 | 1.74 (1.17, 2.63) | 0.006 |
| Hypertension | 2.05 (1.47, 2.86) | <0.001 | 2.01 (1.43, 2.83) | <0.001 |
| **Incident Stroke** |  |  |  |  |
| Higher hs-CRP level | 1.21 (0.80, 1.82) | 0.363 | 1.01 (0.67, 1.55) | 0.942 |
| Hyperhomocysteinemia | 1.68 (1.05, 2.71) | 0.030 | 1.54 (0.96, 2.50) | 0.076 |
| Vitamin D deficiency | 0.92 (0.59, 1.42) | 0.695 | 0.89 (0.57, 1.38) | 0.596 |
| Impaired renal function | 2.26 (1.24, 4.10) | 0.008 | 1.74 (0.94, 3.20) | 0.078 |
| Abnormal ABI | 1.26 (0.70, 2.24) | 0.440 | 1.17 (0.65, 2.10) | 0.593 |
| Obesity | 1.12 (0.63, 1.99) | 0.706 | 1.02 (0.57, 1.82) | 0.952 |
| Central obesity | 1.62 (1.11, 2.35) | 0.012 | 1.45 (0.99, 2.13) | 0.055 |
| Diabetes mellitus | 1.38 (0.87, 2.18) | 0.172 | 1.27 (0.79, 2.02) | 0.319 |
| Hypertension | 1.54 (1.09, 2.18) | 0.013 | 1.46 (1.03, 2.09) | 0.035 |
| **CVD mortality** |  |  |  |  |
| Higher hs-CRP level | 1.32 (0.98, 1.76) | 0.066 | 1.17 (0.87, 1.59) | 0.288 |
| Hyperhomocysteinemia | 1.62 (1.20, 2.20) | 0.002 | 1.47 (1.08, 2.01) | 0.015 |
| Vitamin D deficiency | 1.41 (1.05, 1.89) | 0.021 | 1.36 (1.01, 1.83) | 0.043 |
| Impaired renal function | 1.93 (1.30, 2.87) | 0.001 | 1.72 (1.15, 2.59) | 0.009 |
| Abnormal ABI | 2.40 (1.72, 3.34) | <0.001 | 2.18 (1.56, 3.06) | <0.001 |
| Obesity | 1.47 (1.02, 2.11) | 0.037 | 1.35 (0.94, 1.94) | 0.110 |
| Central obesity | 1.24 (0.96, 1.60) | 0.098 | 1.11 (0.86, 1.44) | 0.430 |
| Diabetes mellitus | 1.90 (1.41, 2.56) | <0.001 | 1.88 (1.39, 2.54) | <0.001 |
| Hypertension | 1.30 (1.02, 1.64) | 0.031 | 1.14 (0.89, 1.47) | 0.301 |

Abbreviation: HR, hazard ratio; 95% CI, 95% confidence interval; CVD, cardiovascular disease; CHD, coronary heart disease; hs-CRP, high-sensitivity C-reactive protein; ABI, ankle-brachial index.

^a^ Model 1: adjusted for sex and age.

^b^ Model 2: adjusted for model 1 plus education level education level, living alone; smoking, alcohol drinking, GDS score, physical activity, energy intake, BMI, history of diabetes and hypertension, and tertiles of DII.

**Table S3**. Sensitivity analysis for associations between tertiles of DII and CVD outcomes.

|  | Tertiles of DII, HR (95% CI) | | | *p*-trend ^a^ |
| --- | --- | --- | --- | --- |
|  | T1 | T2 | T3 |  |
| **Incident CVD** |  |  |  |  |
| Model 2 ^b^ | 1.00 (Ref) | 1.14 (0.85, 1.54) | 1.43 (1.05, 1.96) | 0.020 |
| Model 3 ^c^ | 1.00 (Ref) | 1.13 (0.84, 1.52) | 1.42 (1.04, 1.94) | 0.023 |
| Model 4 ^d^ | 1.00 (Ref) | 1.16 (0.86, 1.58) | 1.47 (1.07, 2.01) | 0.015 |
| **Incident CHD** |  |  |  |  |
| Model 2 | 1.00 (Ref) | 1.02 (0.66, 1.58) | 1.33 (0.85, 2.07) | 0.203 |
| Model 3 | 1.00 (Ref) | 1.02 (0.66, 1.57) | 1.32 (0.84, 2.06) | 0.209 |
| Model 4 | 1.00 (Ref) | 1.05 (0.67, 1.63) | 1.34 (0.85, 2.11) | 0.201 |
| **Incident Stroke** |  |  |  |  |
| Model 2 | 1.00 (Ref) | 1.36 (0.83, 2.21) | 1.56 (0.94, 2.59) | 0.090 |
| Model 3 | 1.00 (Ref) | 1.34 (0.83, 2.19) | 1.56 (0.94, 2.59) | 0.089 |
| Model 4 | 1.00 (Ref) | 1.38 (0.84, 2.26) | 1.63 (0.98, 2.72) | 0.064 |
| **CVD mortality** |  |  |  |  |
| Model 2 | 1.00 (Ref) | 1.27 (0.93, 1.76) | 1.45 (1.03, 2.03) | 0.034 |
| Model 3 | 1.00 (Ref) | 1.27 (0.92, 1.75) | 1.43 (1.02, 2.00) | 0.043 |
| Model 4 | 1.00 (Ref) | 1.30 (0.94, 1.80) | 1.48 (1.05, 2.09) | 0.025 |

Abbreviation: DII, dietary inflammatory index; CVD, cardiovascular disease; CHD, coronary heart disease; HR, hazard ratio; 95% CI: 95% confidence interval; Ref, reference.

^a^ *p* for trend was calculated by treating the median values of DII in tertiles as continuous values in the Cox regression models.

^b^ Model 2: adjusted for sex, age, education level, living alone; smoking, alcohol drinking, GDS score, physical activity, energy intake, BMI, diabetes mellitus and hypertension.

^c^ Model 3: adjusted for Model 2 + antihypertensive, antidiabetic, and lipid-lowering drugs.

^d^ Model 4: adjusted for Model 2, excluding CVD death within two years after baseline (n=41).

**Table S4**. Sensitivity analysis for mediation effects of cardiovascular risk factors on the associations between tertiles of DII and CVD outcomes. ^a^

|  | CVD incidence | |  | CVD mortality | |
| --- | --- | --- | --- | --- | --- |
|  | Proportion mediated, % | *p* |  | Proportion mediated, % | *p* |
| **Model 3** ^b^ |  |  |  |  |  |
| Higher hs-CRP level | 3.00 (−2.12, 14.65) | 0.190 |  | 2.12 (−1.72, 10.56) | 0.266 |
| Hyperhomocysteinemia | 5.77 (1.03, 18.97) | 0.020 |  | 6.19 (1.28, 20.14) | 0.014 |
| Vitamin D deficiency | −0.07 (−10.36, 9.69) | 0.944 |  | −0.09 (−10.29, 8.03) | 0.972 |
| Impaired renal function | 5.03 (1.14, 17.40) | 0.012 |  | 5.83 (1.32, 19.89) | 0.010 |
| Abnormal ABI | 3.33 (0.37, 11.27) | 0.024 |  | 4.63 (0.71, 12.67) | 0.020 |
| Obesity | −0.02 (−2.82, 2.81) | 0.870 |  | −0.33 (−3.75, 1.45) | 0.536 |
| Central obesity | 0.01 (−5.57, 4.90) | 0.988 |  | 0.16 (−3.64, 3.93) | 0.862 |
| Diabetes mellitus | −0.91 (−4.90, 1.17) | 0.306 |  | −0.56 (−4.33, 1.26) | 0.406 |
| Hypertension | −0.73 (−0.63, 3.81) | 0.694 |  | 0.03 (−1.70, 2.40) | 0.896 |
| **Model 4** ^c^ |  |  |  |  |  |
| Higher hs-CRP level | 2.80 (−0.84, 14.42) | 0.132 |  | 2.75 (−1.17, 11.55) | 0.152 |
| Hyperhomocysteinemia | 7.54 (2.62, 22.89) | <0.001 |  | 7.78 (2.48, 27.59) | <0.001 |
| Vitamin D deficiency | 0.08 (−8.17, 6.08) | 0.914 |  | 0.16 (−8.65, 7.01) | 0.936 |
| Impaired renal function | 6.01 (2.02, 19.71) | 0.008 |  | 6.84 (2.24, 23.36) | 0.008 |
| Abnormal ABI | 3.06 (0.37, 9.71) | 0.030 |  | 4.73 (0.76, 12.44) | 0.026 |
| Obesity | −0.17 (−3.85, 1.57) | 0.686 |  | −0.39 (−4.42, 1.30) | 0.474 |
| Central obesity | 0.37 (−5.91. 6.19) | 0.816 |  | 0.01 (−1.85, 1.87) | 0.992 |
| Diabetes mellitus | 0.41 (−5.35, 5.80) | 0.828 |  | 0.39 (−5.58, 5.84) | 0.846 |
| Hypertension | 2.69 (−4.86, 11.35) | 0.426 |  | 0.89 (−2.04, 5.95) | 0.450 |

Abbreviation: DII, dietary inflammatory index; CVD, cardiovascular disease; hs-CRP, high-sensitivity C-reactive protein; ABI, ankle-brachial index.

^a^ Mediated proportions and 95% confidence intervals were presented. Adjusted for sex, age, education level, living alone, smoking, alcohol drinking, physical activity, energy intake.

^b^ Model 3: additionally adjusted for antihypertensive, antidiabetic, and lipid-lowering drugs.

^c^ Model 4: excluding CVD death within two years after baseline (n=41).

**Supplementary Table S5**. Main characteristics of the studies on the association between DII and CVD outcomes.

| **Author, year** | **Study Name, Country/Region** | **Sample size** | **Mean age** | **Follow-up years** | **Diet data**  **collection** | **No. of food parameters** | **DII scores:**  **Mean ± SD**  **or Median (IQR)** | **CVD outcomes:**  **HR or OR (95% CI) (The highest *vs.* lowest category of DII)** | **Mediation analysis (Mediated proportion)** |
| --- | --- | --- | --- | --- | --- | --- | --- | --- | --- |
| - | The Mr. OS and Ms. OS Study, Hong Kong | 3,013 | 72.4 | 5.7 for CVD events;  16.8 for mortality | FFQ | 30 | −0.48 ± 1.46  −0.52 (−1.50, 0.43) | Incident CVD: 1.43 (1.05, 1.96)  CHD: 1.33 (0.85, 2.07)  Stroke: 1.56 (0.94, 2.59)  CVD mortality: 1.45 (1.03, 2.03) | Hyperhomocysteinemia, impaired renal function, and abnormal ABI (3.68% to 7.78%) |
| Liu, 2024 [2] | CHNS, China | 4,822 | 40.8 | 18 | 3-d, 24-h dietary recalls | 28 | −0.8 ± 1.1 | CVD: 1.63 (1.12, 2.39)  Stroke: 1.96 (1.19, 3.21)  MI: 1.45 (0.84, 2.48) | TyG index  (5.90% to 9.35%) |
| Huang, 2024 [3] | NHANES, USA | 13,063 | 47.4 | Cross-sectional | One 24-h dietary recalls | 27 | 1.43 ± 1.86 | Stroke: 1.70 (1.16, 2.50) | No mediation observed for HOMA-IR |
| Xu, 2025 [4] | NHANES, USA | 43,842 | 49.8 | Cross-sectional | Two 24-h dietary recalls | 28 | 1.33 (0.11, 2.40) | CHD: 1.095 (1.024, 1.171) | TyG, VAI, BMI, WtHR, HDL, and GFR may mediate |
| Garcia-Arellano, 2015 [5] | PREDIMED study, Spain | 7,216 | 67 | 4.8 | FFQ | 32 | −0.75 ± 1.53 | CVD: 1.73 (1.15, 2.60) | NA |
| Shivappa, 2018 [6] | MONICA-KORA Cohort, Germany | 1,297 men | 54.5 | 18.2 | 7-d food records | 25 | 0.89 ± 1.38 | CVD mortality: 1.37 (0.89, 2.15)  CHD mortality: 1.25 (0.71, 2.21)  Incident CHD: 1.83 (1.12, 3.01) | NA |
| Khan, 2020 [7] | KoGES_HEXA, South Korea | 162,773 | 52.5 | 7.4 | FFQ | 37 | 0.92 for men  0.89 for women | CVD: 1.32 (1.05, 1.67)  MI: 1.23 (0.94, 1.60)  Stroke: 1.55 (0.99, 2.44) | NA |
| Okada, 2019 [8] | JACC, Japen | 58,782 | 56.2 | 19.3 | FFQ | 26 | 1.02 ± 1.66 | CVD mortality: 1.30 (1.13, 1.49)  CHD mortality: 1.30 (0.96, 1.76)  Stroke mortality: 1.29 (1.05, 1.59) | NA |
| Ganbaatar, 2023 [9] | NIPPON DATA80, Japen | 9,284 | 50 | 29 | 3-d food records | 20 | −0.44 ± 1.14 | CVD mortality: 1.36 (1.15, 1.62)  CHD mortality: 1.71 (1.17, 2.51)  Stroke mortality: 1.33 (1.02, 1.74) | NA |
| Neufcourt, 2016 [10] | SU.VI.MAX study, France | 7,743 | 50 | 11.4 | Six 24-h dietary recalls | 36 | 0.69 ± 1.88 | CVD: 1.15 (0.79, 1.69)  MI: 2.24 (1.08, 4.67)  Stroke: 1.22 (0.56, 2.66) | NA |
| Vissers, 2016 [11] | ALSWH, Australia | 6,972 women | 53 | 11 | FFQ | 25 | 1.42 (2.4) for CVD  1.33 (2.3) for non-CVD | CVD: 1.03 (0.76, 1.42)  IHD: 1.33 (0.86, 2.06)  MI: 1.59 (0.72, 3.52)  Cerebrovascular disease:  0.57 (0.29, 1.15)  Stroke: 0.55 (0.24, 1.26) | NA |
| Li, 2020 [12] | NHS, NHSII and HPFS, USA | 210,145 | 51.0  36.5  54.2 | 24 to 30 | FFQ | 18 food groups ^a^ | Food-based EDIP:  0.04 for NHS; −0.01 for NHSII; 0.06 for HPFS | CVD: 1.38 (1.31, 1.46)  CHD: 1.46 (1.36, 1.56)  Stroke: 1.28 (1.17, 1.39) | NA |

Abbreviation: FFQ, food frequency questionnaire; CVD, cardiovascular disease; CHD, coronary heart disease; MI, myocardial infarction; IHD, ischemic heart disease; ABI, ankle-brachial index; TyG, triglyceride-glucose index; HOMA-IR, homeostasis model assessment of insulin resistance; VAI, visceral adiposity index; BMI, body mass index; WtHR, waist-to-height ratio; HDL, high-density lipoprotein; GFR, glomerular filtration rate.

^a^ The empirical dietary inflammatory pattern (EDIP) was calculated as a weighted sum of 18 food groups, with weights derived from stepwise regression coefficient.

Reference:

[1] Shivappa N, Steck SE, Hurley TG, Hussey JR, Hebert JR. Designing and developing a literature-derived, population-based dietary inflammatory index. Public Health Nutr 2014; 17(8):1689-1696. doi: <http://dx.doi.org/10.1017/S1368980013002115>.

[2] Liu B, Ren X, Tian W. Dietary inflammatory potential and the risk of nonfatal cardiovascular diseases in the China Health and Nutrition Survey. Nutrition 2024; 124:112469.doi: <http://dx.doi.org/10.1016/j.nut.2024.112469>.

[3] Huang R, Lai F, Zhao L, Zhang J, Chen H, Wang S, et al. Associations between dietary inflammatory index and stroke risk: based on NHANES 2005-2018. Sci Rep 2024; 14(1):6704.doi: <http://dx.doi.org/10.1038/s41598-024-57267-9>.

[4] Xu H, Xie P, Liu H, Tian Z, Zhang R, Cui M. The relationship between dietary inflammatory index in adults and coronary heart disease: from NHANES 1999-2018. Front Nutr 2025; 12:1564580.doi: <http://dx.doi.org/10.3389/fnut.2025.1564580>.

[5] Garcia-Arellano A, Ramallal R, Ruiz-Canela M, Salas-Salvado J, Corella D, Shivappa N, et al. Dietary Inflammatory Index and Incidence of Cardiovascular Disease in the PREDIMED Study. Nutrients 2015; 7(6):4124-4138.doi: <http://dx.doi.org/10.3390/nu7064124>.

[6] Shivappa N, Schneider A, Hebert JR, Koenig W, Peters A, Thorand B. Association between dietary inflammatory index, and cause-specific mortality in the MONICA/KORA Augsburg Cohort Study. Eur J Public Health 2018; 28(1):167-172.doi: <http://dx.doi.org/10.1093/eurpub/ckx060>.

[7] Khan I, Kwon M, Shivappa N, Hebert JR, Kim MK. Positive Association of Dietary Inflammatory Index with Incidence of Cardiovascular Disease: Findings from a Korean Population-Based Prospective Study. Nutrients 2020; 12(2):588.doi: <http://dx.doi.org/10.3390/nu12020588>.

[8] Okada E, Shirakawa T, Shivappa N, Wakai K, Suzuki K, Date C, et al. Dietary Inflammatory Index Is Associated with Risk of All-Cause and Cardiovascular Disease Mortality but Not with Cancer Mortality in Middle-Aged and Older Japanese Adults. J Nutr 2019; 149(8):1451-1459.doi: <http://dx.doi.org/10.1093/jn/nxz085>.

[9] Ganbaatar G, Okami Y, Kadota A, Ganbaatar N, Yano Y, Kondo K, et al. Association of Pro-Inflammatory Diet with Long-Term Risk of All-Cause and Cardiovascular Disease Mortality: NIPPON DATA80. J Atheroscler Thromb 2024; 31(3):326-343.doi: <http://dx.doi.org/10.5551/jat.64330>.

[10] Neufcourt L, Assmann KE, Fezeu LK, Touvier M, Graffouillere L, Shivappa N, et al. Prospective Association Between the Dietary Inflammatory Index and Cardiovascular Diseases in the SUpplementation en VItamines et Mineraux AntioXydants (SU.VI.MAX) Cohort. J Am Heart Assoc 2016; 5(3):e002735.doi: <http://dx.doi.org/10.1161/JAHA.115.002735>.

[11] Vissers LET, Waller MA, van der Schouw YT, Hebert JR, Shivappa N, Schoenaker DAJM, et al. The relationship between the dietary inflammatory index and risk of total cardiovascular disease, ischemic heart disease and cerebrovascular disease: Findings from an Australian population-based prospective cohort study of women. Atherosclerosis 2016; 253:164-170.doi: <http://dx.doi.org/10.1016/j.atherosclerosis.2016.07.929>.

[12] Li J, Lee DH, Hu J, Tabung FK, Li Y, Bhupathiraju SN, et al. Dietary Inflammatory Potential and Risk of Cardiovascular Disease Among Men and Women in the U.S. J Am Coll Cardiol 2020; 76(19):2181-2193.doi: <http://dx.doi.org/10.1016/j.jacc.2020.09.535>.
